# Lightweight design of running prosthetic feet using honeycomb sandwich composites: a comparative FEA study

**DOI:** 10.3389/fbioe.2026.1785315

**Published:** 2026-04-02

**Authors:** Yunis Akkaş, Serap Alsancak

**Affiliations:** Department of Orthotics and Prosthetics, Faculty of Health Sciences, Ankara University, Ankara, Türkiye

**Keywords:** finite element analysis, honeycomb composite, ISO 10328, lightweight design, mechanical performance, running prosthetic foot

## Abstract

**Introduction:**

Honeycomb sandwich composites are widely employed in aerospace and structural applications due to their high specific strength and efficient energy storage and return, offering a promising design alternative for running prostheses.

**Methods:**

This study investigates the mechanical performance of a running prosthetic foot designed with an aluminum honeycomb core (AMS 3711) and carbon fiber skins, comparing it against a conventional solid epoxy-carbon laminate. A finite element model was developed in ANSYS following ISO 10328 boundary and loading conditions. The analysis evaluated six design configurations, transitioning from a solid reference blade to optimized sandwich structures. Static analyses were performed at two gait-cycle positions defined by ISO 10328 to evaluate mass, deformation, equivalent stress, strain energy, and safety factors across varying core and surface-layer thicknesses under constant total volume.

**Results:**

The results demonstrated that the optimized sandwich design achieved a 63.9% reduction in distal mass (from 1.33 kg to 0.48 kg) compared to the solid counterpart. Contrary to the typical trade-off between lightweighting and energy capacity, the increased compliance of the sandwich structure resulted in a 57.4% increase in strain energy storage. Although the transition to a cellular core initially reduced the safety margin, geometric optimization of the core thickness recovered structural integrity, achieving a safety factor of 1.95, well above the recommended threshold of 1.5.

**Conclusion:**

These findings indicate that honeycomb sandwich architectures offer a superior alternative to solid laminates for running prostheses, simultaneously enhancing energy return efficiency and minimizing structural mass without compromising safety.

## 1 Introduction

Honeycomb sandwich structures are widely used in aerospace, automotive, and marine engineering because of their high strength-to-weight ratio, bending stiffness, and geometric versatility (Guo et al., 2024; Zhang et al., 2023). Composed of a lightweight core bonded between stiff face sheets, these structures enable designers to tune mechanical performance by precisely adjusting core geometry and skin material (Xiao et al., 2024). Honeycomb cores such as nomex and aluminum provide high compressive and shear strength, allowing these structures to withstand static, cyclic, and impact loads at low mass while effectively dissipating or storing energy (Ayanoglu et al., 2021; Hou et al., 2011). The mechanical behavior of sandwich structures is largely determined by the interaction between the core and skin layers; while increasing core thickness generally enhances bending stiffness and load-carrying capacity, excessive thickness may compromise weight efficiency (Zhou et al., 2006; Georges et al., 2024). Skin stiffness and loading mode further influence this balance, necessitating carefully optimized core–skin configurations (Liu et al., 2019).

The favorable structural characteristics of honeycomb composites—specifically high strain-energy capacity, controlled deformation, and minimal mass—align closely with the biomechanical requirements of running prosthetic feet. These devices must withstand high-frequency and high-impact loads while enabling efficient energy storage and return. During mid-stance, elastic deformation stores strain energy that is released during push-off, generating propulsion, aiming to reproduce the function of the biological foot–ankle complex (Tabucol et al., 2021; Waetjen et al., 2012; Hobara et al., 2013; Grabowski et al., 2010). Optimal performance depends on achieving a functional balance between rigidity and flexibility: excessive stiffness restricts natural running mechanics and reduces energy return, whereas insufficient stiffness compromises stability and force transmission (Hobara et al., 2015; Mahm et al., 2016). Prosthetic mass is an additional critical factor, as lighter components improve swing-phase dynamics and sprinting performance, motivating the widespread use of carbon-fiber composites due to their favorable strength-to-weight properties and durability (Talla et al., 2022). However, traditional monolithic carbon-fiber laminates limit the ability to independently tailor bending stiffness, strain-energy behavior, and mass distribution.

Finite element analysis (FEA) has been extensively used to study the mechanical performance of running prosthetic feet. Recent advancements in the field include dynamic FE analyses and sophisticated simulation frameworks that incorporate user-specific kinematics and ground reaction forces to evaluate blades under realistic running conditions (Rigney et al., 2016; Barattini et al., 2025). While these dynamic models provide critical insights into *in-vivo* gait mechanics, standardized static FEA remains an essential foundation for benchmarking novel material architectures and structural geometries. For instance, Krishna et al. evaluated stress and deformation patterns across different materials, highlighting the need for structural optimization in prosthetic blade design (Krishna et al., 2023). Similarly, Siddiqui et al. further examined the static behavior of blades manufactured from aluminum alloys, stainless steel, titanium, and carbon fiber under various physiological loads, consistently demonstrating that carbon-fiber blades exhibit superior deformation control and favorable mechanical response (Siddiqui et al., 2023a; Siddiqui et al., 2023b). Subsequent computational studies comparing various geometric configurations confirmed that carbon composites outperform conventional metallic alternatives regarding stiffness and stress distribution (Ismail et al., 2020). While these studies effectively demonstrate the utility of FEA for quantifying deformation, equivalent stress, strain energy, and safety factors, they predominantly focus on monolithic laminate constructions. No existing computational research systematically investigates honeycomb sandwich architectures or evaluates how variations in core thickness affect stiffness–weight balance, strain-energy behavior, or mechanical safety under physiologically relevant loading conditions.

These limitations underscore the need for structural concepts that transcend traditional laminate approaches by exploring tunable internal architectures capable of enhancing energy-storage capacity, reducing mass, and optimizing deformation behavior. Honeycomb sandwich structures, despite their demonstrated advantages in aerospace and automotive engineering, have not yet been assessed as a viable design option for high-performance running prostheses.

The aim of this study is to evaluate the feasibility of integrating honeycomb sandwich composite structures into the design of running prosthetic feet. To address the identified research gap, this study systematically analyzes various honeycomb core thicknesses and compares their mechanical performance against a reference prepreg carbon composite configuration. Finite element simulations, conducted under ISO 10328 loading conditions, were utilized to assess deformation behavior, strain-energy capacity, equivalent stress distribution, safety factors, and mass efficiency. Through this comparative framework, the study investigates whether honeycomb-based architectures can provide a more favorable stiffness-to-weight ratio for high-performance running prostheses.

## 2 Materials and methods

This study employed a finite element analysis framework to evaluate the mechanical behavior of honeycomb sandwich prosthetic foot designs benchmarked against a standard prepreg carbon composite configuration. All modeling steps, material definitions, geometric parameters, boundary conditions, and analysis procedures were maintained consistently across simulations to ensure direct comparability between different core thicknesses. The methodology is detailed in the following subsections.

### 2.1 Geometry modeling

The geometry of the running prosthetic foot was developed based on the characteristic C-shaped profile typical of commercially available running blades designed for sprinting applications. Unlike conventional prosthetic feet intended for everyday walking, running blades feature a continuous curved structure without a distinct heel component, allowing elastic deformation along the entire length of the blade. In this study, the blade profile—including its curvature radius, longitudinal arc, and varying width along the span—was recreated by taking manual measurements from an existing physical prosthetic running blade to generate a precise technical drawing, which was subsequently converted into a three-dimensional Computer-Aided Design (CAD) model. While commercial running blades often feature non-constant (tapered) thickness distributions to tune localized stiffness, a constant total thickness was deliberately assumed for all models in this study. This idealized geometric constraint was necessary to strictly isolate the influence of the honeycomb core thickness on the overall mechanical behavior, ensuring that variations in stiffness and mass were solely attributable to the internal sandwich architecture rather than external geometric tapering. All geometric modeling was performed using SpaceClaim (ANSYS Inc., Canonsburg, PA, USA). The model was initially generated as a surface representation, after which solid regions corresponding to the composite skin layers and the internal honeycomb core were defined. For honeycomb-integrated configurations, the core cavity was dimensioned to incorporate varying honeycomb thicknesses, while maintaining the external blade envelope identical across all models. This ensured that all configurations shared the same global shape and total volume, allowing a controlled comparison of mechanical behavior influenced solely by core thickness variation.

### 2.2 Material properties

The finite element simulations incorporated two distinct material systems: (1) an Epoxy Carbon Woven composite utilized for the structural skin layers, and (2) an aluminum honeycomb structure serving as the core. The mechanical properties of the Epoxy Carbon Woven composite were adopted from the standard ANSYS Engineering Data Library (Ashby, 2021). The material parameters of the aluminum honeycomb core were obtained from technical datasheets provided by Toray Advanced Composites (Toray Advanced Composites, 2025).

The composite skins were modeled as linear-elastic orthotropic materials, with directional elastic moduli, shear moduli, and Poisson’s ratios assigned in accordance with the standard ANSYS material library. Conversely, the honeycomb core was modeled as an equivalent orthotropic continuum, defined based on the stabilized out-of-plane compression and shear properties reported in the manufacturer’s technical datasheet. The material parameters used in the simulations—including density, elastic modulus, shear properties, and strength values—are summarized in Tables 1, 2.

**TABLE 1:** Material properties of the Epoxy Carbon Woven composite (Ashby, 2021).

Material Property	Material Properties
*Ρ* (g·cm^−3^)	1.48
*E*_1_ (MPa)	91,820
*E*_2_ (MPa)	91,820
*E*_3_ (MPa)	9,000
ν_12_	0.05
ν_23_	0.02
ν_13_	0.02
*G*_12_ (MPa)	3,600
*G*_23_ (MPa)	3,600
*G*_13_ (MPa)	3,600
Tensile strength X direction (MPa)	829
Tensile strength Y direction (MPa)	829
Tensile strength Z direction (MPa)	50
Compressive strength X direction (MPa)	−439
Compressive strength Y direction (MPa)	−439
Compressive strength Z direction (MPa)	−140
Shear strength XY (MPa)	120
Shear strength YZ (MPa)	50
Shear strength XZ (MPa)	50

**TABLE 2:** Material properties of the Aluminum Honeycomb Core (AMS 3711) (Toray Advanced Composites, 2025).

Material Property	Material Properties
*Ρ* (g·cm^−3^)	0.13
*E*_1_ (MPa)	1
*E*_2_ (MPa)	1
*E*_3_ (MPa)	2,413
ν_12_	0.3
ν_23_	0.001
ν_13_	0.001
*G*_12_ (MPa)	1
*G*_23_ (MPa)	931
*G*_13_ (MPa)	931
Bare compressive strength T direction (MPa)	10.75
Plate shear Strength L direction (MPa)	5
Plate shear strength W direction (MPa)	5

**FIGURE 1:**
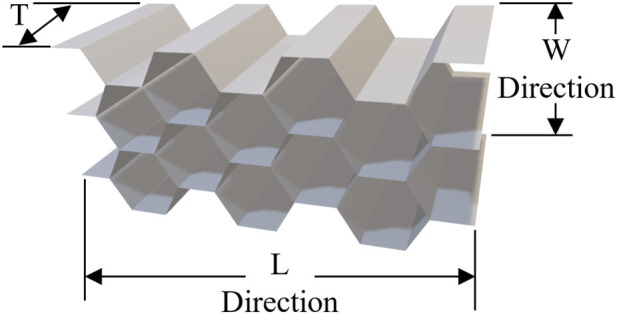
Orthotropic directions of the Aluminum honeycomb core.

The orthotropic directions of the honeycomb material (L, W, and T) were assigned in accordance with the manufacturer’s definition, as illustrated in Figure 1, ensuring consistent orientation during finite element modeling across all configurations.

Aluminum honeycomb was specifically selected over alternative lightweight core materials due to its superior transverse shear modulus, high cyclic fatigue resistance, and geometric conformability. These characteristics are critical for enduring the severe bending moments of a running blade while maintaining a high stiffness-to-weight ratio. According to the product datasheet provided by Toray for the AAA-8.1-1/8-20N-5052 grade, the core is manufactured using the standard expansion method. It consists of regular hexagonal cells with a nominal cell size of 3.175 mm (1/8 inch) and an aluminum (5052 alloy) nonperforated foil wall thickness of 0.05 mm (0.002 inch) (Toray Advanced Composites, 2025). In a realistic manufacturing scenario, this sandwich structure would be fabricated via a co-curing process. The structural integrity between the prepreg carbon skins and the aluminum honeycomb core would be secured by interposing an epoxy-based structural film adhesive at the interface, ensuring robust shear load transfer and preventing localized delamination under dynamic running conditions.

### 2.3 Honeycomb configurations

The running blade was modeled as a sandwich composite structure in which carbon composite skin layers encapsulate an aluminum honeycomb core to systematically investigate the influence of core architecture on mechanical performance, five distinct honeycomb configurations were generated with core thicknesses of 6 mm, 8 mm, 10 mm, 12 mm and 14 mm. Throughout these variations, the total blade thickness was strictly maintained at a constant 20 mm. Thus, the thickness of the enclosing carbon skin layers was adjusted inversely to accommodate the varying core dimensions, ensuring that the external volume remained identical across all simulations.

Because the overall thickness was fixed, the number of carbon woven layers was adjusted inversely to the core thickness. Each carbon woven ply has a nominal thickness of 0.25 mm, and additional plies were added to compensate for thinner cores. Thus, configurations with smaller cores incorporated more carbon plies, while those with thicker cores required fewer plies. All layups employed a symmetric laminate sequence. The stacking sequence was defined by orienting the primary warp direction of the woven plies, denoted in standard woven notation as [(0/90), (+45/−45)]s. This sequence was repeated on both sides of the mid-plane to prevent bending–twist coupling and ensure balanced mechanical behavior throughout the curved geometry.

**FIGURE 2:**
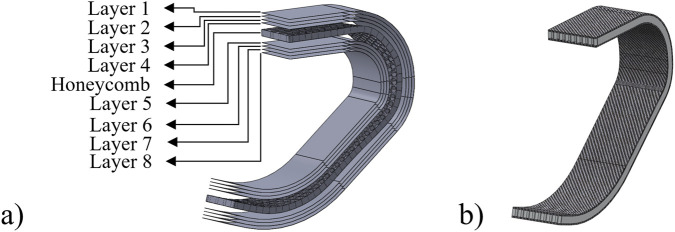
Sandwich composite structure of the running blade; **(a)** Exploded view showing carbon plies and honeycomb core, **(b)** Assembled 20 mm sandwich configuration.

**FIGURE 3:**
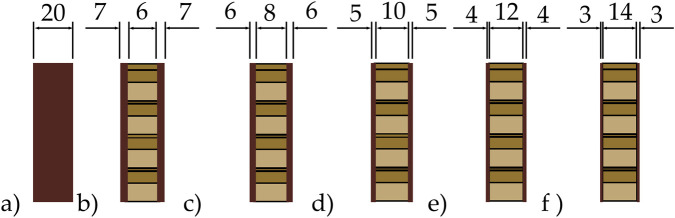
Cross-sectional thickness configurations: **(a)** Solid 20 mm carbon model; **(b-f)** Sandwich designs with 6-, 8-, 10-, 12-, and 14-mm honeycomb cores and adjusted carbon skin thickness.

The internal sandwich structure, including the honeycomb placement and the distribution of composite layers, is illustrated in Figure 2, which presents both the exploded laminate scheme and the fully assembled blade structure. To further clarify the controlled comparison approach used in this study, Figure 3 shows the cross-sectional thickness distributions of all configurations. This schematic highlights how the 20 mm total thickness was preserved by adjusting skin thickness in response to the different honeycomb core dimensions. Together, Table 3 clearly demonstrate the construction of the sandwich composite blade and the methodology used to isolate the influence of honeycomb core thickness on structural performance.

**TABLE 3:** Definition of simulated cases and geometric configurations.

Case ID	Configuration type	Core thickness (*t*_*c*_), mm	Skin thickness per face (*t*_*f*_), mm	Number of plies per face	Total thicknes mm
Case 1	Solid laminate	0	20	40	20
Case 2	Sandwich structure	6	7	28	20
Case 3	Sandwich structure	8	6	24	20
Case 4	Sandwich structure	10	5	20	20
Case 5	Sandwich structure	12	4	16	20
Case 6	Sandwich structure	14	3	12	20

### 2.4 Finite element setup

To ensure the numerical accuracy and reliability of the finite element results, a rigorous mesh sensitivity analysis (convergence study) was conducted. The primary objective of this procedure was to eliminate discretization errors and establish a grid-independent solution, ensuring that the calculated stress and deformation values are governed by the physics of the problem rather than the number of elements.

The computational domain was discretized using high-quality quadrilateral (Quad) shell elements. A structured mapped meshing technique was employed to maintain uniform element aspect ratios, particularly along the curved sections of the blade where stress gradients are critical.

**FIGURE 4:**
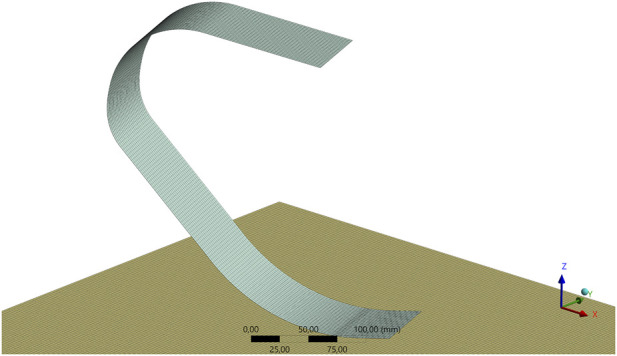
Finite element mesh of the prosthetic running blade featuring a structured quadrilateral element distribution.

Following the convergence analysis, the element size was iteratively refined until the deviation in key output parameters (vertical displacement (directional deformation) and equivalent stress) stabilized. The final optimized mesh configuration, selected for all subsequent analyses, consists of 30,936 elements and 188,247 nodes. Figure 4 illustrates the high-fidelity structured mesh applied to the blade geometry.

The complex lamination schedule of the prosthetic blade was implemented using the Ansys Composite PrepPost (ACP) module, facilitating a high-fidelity, manufacturing-centric modeling approach. In contrast to simplified homogenous material assignments, ACP permits a discrete layer-by-layer definition of the composite structure, thereby accurately capturing the orthotropic mechanical response of the stack-up. Two distinct “Fabric” definitions were established: (1) Epoxy Carbon Woven with a nominal ply thickness of 0.25 mm, and (2) Aluminum Honeycomb core with variable thickness parameters (6–14 mm) corresponding to the specific study cases. For all configurations, a symmetric laminate schedule was strictly enforced. The final composite definition was mapped onto the finite element mesh as shell section data, preserving the computational efficiency of the shell elements while accurately representing the through-thickness properties of the sandwich structure.

The interaction between the prosthetic blade’s plantar surface (contact body) and the ground plate (target body) was modeled using a “Frictionless” contact formulation. This non-linear contact type allows for the separation of surfaces (lift-off) and unrestricted tangential sliding, while strictly preventing penetration in the normal direction.

Although friction exists in real-world gait scenarios, a frictionless assumption was adopted in this study to provide a conservative estimate of the structural deformation. By allowing the distal end of the blade to slide and splay freely under vertical loading, any artificial stiffening effects caused by friction-induced constraints were eliminated. This ensures that the resulting displacement and internal stresses represent a “worst-case” condition for bending compliance, verifying that the laminate design can withstand loads without relying on traction forces to maintain structural stability.

The contact behavior was controlled using the Augmented Lagrange formulation to minimize penetration. To further facilitate convergence, the interface stiffness was adaptively updated throughout the non-linear solution process.

### 2.5 ISO 10328 loading conditions

Structural verification of the prosthetic blade was conducted in accordance with the ISO 10328:2016 international standard. Considering the demographics of the target user population and the objective of lightweight structural optimization, Test Load Level P4 (m ≤ 80 kg) was selected as the reference loading category. Within this framework, the finite element analyses were performed using the ultimate static test forces (FSU) specified for the loading level. Accordingly, the blade was subjected to a vertical load of 3098 N under Loading Condition I to simulate the toe-off phase, and 2717 N under Loading Condition II to simulate the heel-strike phase.

To systematically evaluate the blade’s performance across critical phases of the gait cycle, two distinct loading configurations defined in the standard were simulated:

- Condition I (Forefoot Loading): This configuration replicates the “toe-off” (propulsion) phase, where the load is concentrated at the distal end of the blade. This scenario represents the most critical condition for assessing the maximum bending moment and the strain energy storage capacity of the structure.
- Condition II (Heel Loading): This configuration simulates the “heel-strike” (initial contact) phase. Although the running blade design lacks a defined heel component, this load case is essential for verifying the structural integrity of the proximal curvature under impact loads experienced at the moment of ground contact.

While physical ISO test setups typically apply load along a specific “Load Line” relative to knee and ankle centers, the boundary conditions in this finite element study were defined to simulate the blade being compressed against a stationary surface. The ground plate was modeled as a rigid body constrained by a Fixed Support, fully restricting all degrees of freedom. Correspondingly, the test load—with magnitude determined by the specific ISO loading condition—was applied as a vertical force vector to the proximal adapter interface (the superior surface of the blade).

To faithfully replicate the specific load line geometry mandated by ISO 10328 for Conditions I and II, the spatial alignment of the blade and the rigid ground plate was strictly defined using the global coordinate system. Unlike conventional prosthetic feet, running blades are positioned by aligning the setup with the specific effective top and bottom load application coordinates provided by the standard. This macroscopic geometric positioning ensured that upon applying the vertical load to the proximal adapter, the resultant ground reaction force vector aligned precisely with the standard’s spatial specifications for both the toe-off and heel-strike scenarios.

**FIGURE 5:**
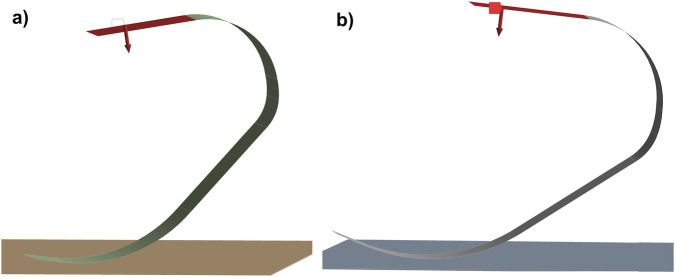
Finite element setup simulating the ISO 10328 Load Line **(a)** Condition I, **(b)** Condition II.

The finite element implementation of the ISO 10328 load line configuration is illustrated in Figure 5. Specifically, Figure 5a depicts Condition I (Forefoot Loading), whereas Figure 5b represents Condition II (Heel Loading). In both scenarios, the precise loading vector prescribed by the standard was achieved by adjusting the angular orientation of the ground plate relative to the vertically loaded blade.

### 2.6 Output metrics

To systematically evaluate the structural efficiency and biomechanical potential of the proposed honeycomb-reinforced designs, four key performance indicators were derived from the finite element simulations.

#### 2.6.1 Mass (lightweighting potential)

The total mass of each configuration was evaluated to quantify the weight reduction achieved by substituting solid carbon laminate layers with the lightweight aluminum honeycomb core. Minimizing distal mass is paramount in prosthetic design to reduce the moment of inertia, thereby limiting user fatigue and metabolic cost during the swing phase of running.

#### 2.6.2 Vertical displacement (stiffness characteristics)

The maximum deflection (δ) under the prescribed load was recorded as an inverse indicator of structural stiffness. For running blades, deformation is a prerequisite for effective shock absorption; however, excessive flexibility can compromise gait stability. The vertical displacement at the toe was analyzed to ensure the compliance remained within a functional biomechanical range.

#### 2.6.3 Strain energy

The total elastic strain energy (U) stored within the entire assembly at peak loading was computed. In the context of passive prosthetic feet, which lack active propulsion, strain energy serves as the primary metric for “energy return”. A higher energy storage capacity implies that the blade can storage more kinetic energy during the stance phase and release it more efficiently during the push-off phase.

#### 2.6.4 Failure criteria and safety factors

To predict the structural integrity of the hybrid assembly, distinct failure criteria were applied to the composite skins and the cellular core, reflecting their unique failure mechanisms:

- Carbon Fiber Skins: The Tsai-Wu failure criterion was employed for the orthotropic laminate layers. This interactive criterion accounts for the combined effects of multi-axial stresses (tension, compression, and shear). The safety factor (SF_skin_) is defined as the inverse of the failure index (FI):
$$SF_{skin}=\frac{1}{{FI}_{Tsai-Wu}}$$
- Aluminum Honeycomb Core: Since the failure mode of the core is location-dependent, a multi-modal evaluation approach was adopted:
  ○ Core Shear: In the bending regions, the core acts primarily to transfer shear loads between facesheets. Therefore, the Maximum Shear Stress criterion was applied. The safety factor (SF_shear_) is calculated based on the ratio of the material’s allowable shear strength (in the ribbon direction) to the maximum calculated shear stress:
$$SF_{Core}=\frac{\tau_{allowable}}{\tau_{\max}}$$
  ○ Core Crushing: At the localized load application points (e.g., ground contact and adapter interface), the core is subjected to compressive forces. To assess the risk of crushing, the Flatwise Compressive Strength criterion was used. The safety factor (SF_comp_) is derived from the ratio of the stabilized compressive strength to the maximum normal stress in the Z-direction:
$$SF_{Comp}=\frac{\sigma_{Comp_{L}imit}}{\sigma_{z_{m}ax}}$$

The reported Global Safety Factor for each case represents the minimum value observed across the entire component (*SF*_*global*_ = min (*SF*_*skin*_, *SF*_*core*_)). A safety factor below 1.0 indicates predicted failure.

### 2.7 Analytical verification of the FE model

The fidelity of the finite element model was verified analytically using Classical Sandwich Beam Theory. To confirm the accuracy of the element formulation and material definitions, the equivalent stress results of a representative straight cantilever beam case were compared against theoretical calculations.

A beam model with identical laminate properties to the prosthetic blade was defined. According to the “Technical Sandwich” assumptions—where the core is soft (*E*_*C*_ *< E*_*F*_) and face sheets are thin—the equivalent flexural rigidity (EI_eq_) of the section is dominated by the face sheets’ Steiner term and was calculated as follows (Öchsner et al., 2024):

$$EI_{eq}\approx \frac{E_{f}\cdot b\cdot t_{f}\cdot h_{c}^{2}}{2}$$

Where Ef is the elastic modulus of the carbon skins, b is the width, tf is the skin thickness (Δ*h* ^*F*^) and hc is the distance between the centroids of the face sheets (*h*_*c*_ *= t*_*core*_ *+ t*_*f*_).

The theoretical normal stress (*σ*_*theory*_) on the outer skin was derived using the simplified stress formula for technical sandwich beams (Öchsner et al., 2024):

$$\sigma_{theory}\approx \frac{M}{b\cdot t_{f}\cdot h_{c}}$$

## 3 Results

The structural performance of the proposed prosthetic foot designs was evaluated under the Ultimate Static Test Forces prescribed by the ISO 10328:2016 standard. Based on the targeted user mass, Test Loading Level P4 (m ≤ 80 kg) was selected as the reference loading category. The simulations were conducted under the specific ultimate load values (Fsu) defined for the lower test level:

- Condition I (Forefoot/Toe-off): A vertical load of 3098 N was applied.
- Condition II (Heel/Heel-strike): A vertical load of 2717 N was applied.

To facilitate a systematic comparative analysis, the six design configurations illustrated previously in Figure 3 are designated as Case 1 through Case 6. As detailed in Table 3, Case 1 represents the reference solid carbon blade, while Cases 2–6 correspond to the honeycomb-reinforced sandwich designs with incrementally increasing core thicknesses, all maintaining a constant total profile thickness of 20 mm.

### 3.1 Mass reduction vs. stiffness stability

**FIGURE 6:**
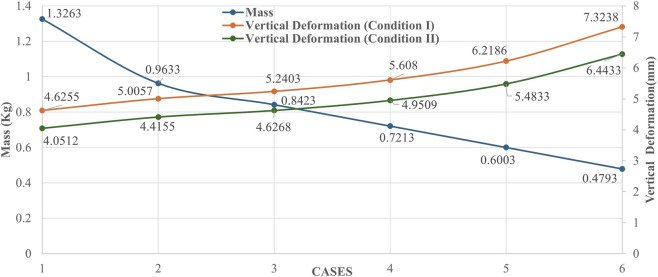
Comparative analysis of structural mass and vertical deformation across the six design configurations.

One of the primary objectives of this study was to minimize the distal mass of the prosthetic blade to reduce the metabolic cost of running, while maintaining structural stability. The trade-off between lightweighting and mechanical compliance is visualized in Figure 6. The reference solid carbon design (Case 1) exhibits a total mass of 1.326 kg. In contrast, the optimized sandwich configuration with the thickest core (Case 6) reduced the mass to 0.479 kg.

This transition corresponds to a substantial weight reduction of approximately 63.9%. Regarding the stiffness characteristics, the introduction of the cellular core resulted in a controlled increase in flexibility under both loading scenarios. Under Condition I, the vertical deformation increased from 4.63 mm in the solid reference design to 7.32 mm in the 14-mm core configuration. Under Condition II, a similar increase was observed, with deformation rising from 4.05 mm to 6.44 mm.

To further analyze the deflection behavior, the directional deformation along the vertical loading axis (Z-axis) was isolated. As presented in Table 4, the vertical displacement values closely align with the vertical deformation magnitudes. For the reference solid blade (Case 1), the Z-axis deformation was recorded as −4.42 mm under forefoot loading. In the most compliant sandwich configuration (Case 6), this value reached −7.01 mm.

**TABLE 4:** Directional deformation in the vertical loading axis (Z-axis) for all cases.

Design Case	Vertical deformation	
	Condition I	Condition II
Case 1	−4.4228	−3.5068
Case 2	−4.7858	−3.851
Case 3	−5.0115	−4.0377
Case 4	−5.3664	−4.3198
Case 5	−5.9552	−4.7783
Case 6	−7.0159	−5.5958

### 3.2 Stress distribution on carbon skins

The structural integrity of the prosthetic blades was assessed by monitoring the Equivalent (von-Mises) Stress distribution across the composite laminates. Table 5 summarizes the global maximum stress values recorded within the structure for each design configuration.

**TABLE 5:** Maximum equivalent (von-Mises) stress values observed for all design configurations under Condition I and Condition II loading.

Design Case	Equivalent stress (MPa)	
	Condition I	Condition II
Case 1	128.64	113.65
Case 2	131.32	115.83
Case 3	136.61	121.10
Case 4	146.43	131.00
Case 5	163.96	148.02
Case 6	197.20	179.03

As expected, geometric optimization combined with a reduction in the load-bearing cross-section led to increased stress concentrations. Under the critical Condition I loading, the peak equivalent stress within the blade structure increased from 128.64 MPa in Case 1–197.20 MPa in the 14-mm honeycomb configuration (Case 6). This represents a 53.3% increase in peak stress. A similar increase was observed for Condition II loading scenario, where stress values increased from 113.65 MPa to 179.03 MPa.

**FIGURE 7:**
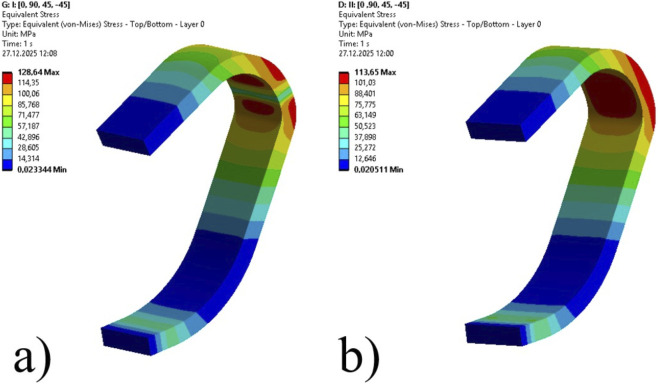
Equivalent (von Mises) stress distribution contours for Case 1: **(a)** Condition I (Max: 128.64 MPa), **(b)** Condition II (Max: 113.65 MPa).

**FIGURE 8:**
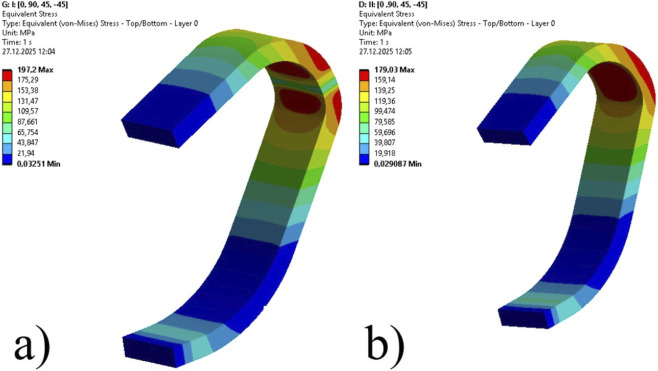
Equivalent (von Mises) stress distribution for the optimized sandwich blade (Case 6). **(a)** Condition I (Max: 197.20 MPa), **(b)** Condition II (Max: 179.03 MPa).

Figures 7, 8 illustrates the stress contours for the limiting cases. As observed in Figure 7, the solid laminate exhibits a broad stress distribution. In contrast, the sandwich structure in Figure 8 shows that while the stress magnitude increased, the distribution pattern remained consistent, with peak stresses concentrated at the distal curvature.

To verify the reliability of the Finite Element model, an analytical validation was conducted based on standard Sandwich Beam Theory for the most critical design configuration (Case 6). Under the Condition I loading, the theoretical maximum bending stress on the outer face sheet was calculated as 192.86 MPa. Comparing this analytical baseline with the numerical result of 197.20 MPa, the deviation is found to be only 2.2%

### 3.3 Honeycomb core mechanics

In sandwich composites, the core material acts as the shear web, transferring loads between the stiff carbon face sheets. The mechanical reliability of the core was evaluated by analyzing the Transverse Shear Stress and the Through-Thickness Normal Stress (*σ*_*z*_). Table 6 presents the peak values recorded for the honeycomb-reinforced configurations (Cases 2–6).

**TABLE 6:** Maximum shear stress and through-thickness normal stress (σz) developed in the honeycomb core.

Design Case	Max shear stress		Z - normal stress	
	Condition I	Condition II	Condition I	Condition II
Case 2	3.01	2.64	−2.65	−4.49
Case 3	2.88	2.53	−2.53	−4.16
Case 4	2.76	2.42	−2.42	−3.86
Case 5	2.66	2.33	−2.33	−3.60
Case 6	2.57	2.25	−2.28	−3.35

Under Loading Condition I, which induces maximum bending moments, the core experienced the highest shear demand. The interaction between the core and the face sheets was further analyzed by examining the Z-directional normal stress. Positive values typically indicate tensile forces that can lead to skin-core debonding (delamination), while negative values indicate compressive crushing. As detailed in Table 6, all recorded normal stress values were consistently negative across all cases and loading conditions. In addition to normal stresses, the transverse shear stresses reported in Table 6 dictate the shear demand at the skin-core interface. Excessive interfacial shear can lead to sliding or debonding between the carbon plies and the aluminum honeycomb, which is a critical failure mode in sandwich structures. However, the maximum shear stress observed across all configurations peaked at 3.01 MPa (Case 2). Because this value remains significantly lower than the typical shear strength of structural epoxy film adhesives, which generally exceed 30 MPa (Kinloch, 1987; Banea and da Silva, 2009) the risk of interfacial sliding or macroscopic debonding is considered minimal.

### 3.4 Strain energy capacity

**FIGURE 9:**
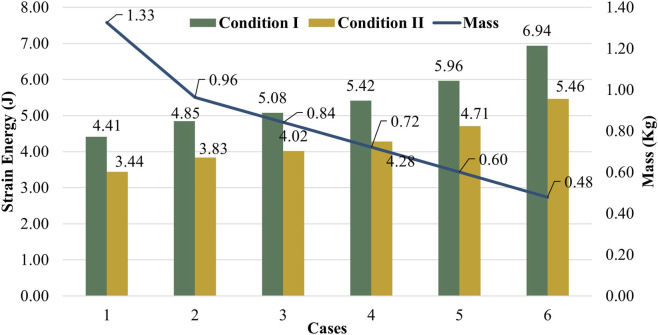
Dual-axis comparison of Strain Energy (bars) and Structural Mass (line).

The ability of a prosthetic blade to store and release elastic energy is critical for metabolic efficiency during running. The strain energy capacity of each design configuration was evaluated under both loading conditions and is correlated with the structural mass in Figure 9.

Contrary to the typical trade-off observed in lightweight structures, the results reveal a favorable inverse relationship between mass and energy storage. As the honeycomb core thickness increased and the carbon skin volume decreased, the strain energy capacity exhibited a significant upward trend. Under Loading Condition I, the stored strain energy increased from 4.41 J in Case 1–6.94 J in Case 6, corresponding to a 57.4% increase in energy storage capability.

### 3.5 Structural safety factors

The minimum safety factors (SF) for each design configuration were calculated based on the failure criteria of the constituent materials. Table 7 presents the computed safety margins under both ISO 10328 loading scenarios.

**TABLE 7:** Minimum structural safety factors observed under ISO 10328 loading conditions.

Design Case	Safety factors	
	Condition I	Condition II
Case 1	4.11	4.65
Case 2	1.66	1.90
Case 3	1.73	1.98
Case 4	1.81	2.06
Case 5	1.88	2.15
Case 6	1.95	2.22

For Case 1, the safety factor was determined to be 4.11 under Condition I loading. Following the transition to the sandwich architecture, the minimum safety factor was recorded as 1.66 in the thinnest core configuration (Case 2).

As the honeycomb core thickness increased from 6 mm (Case 2) to 14 mm (Case 6), the safety factor exhibited a linear increase, reaching 1.95 in the final configuration. Under heel loading (Condition II), the safety factors were consistently higher compared to the forefoot condition, ranging from 4.65 in the reference design to 2.22 in Case 6.

Because the running blade is a multi-material sandwich structure, relying on a single, generalized FEA safety factor output can be misleading. Therefore, the Global Safety Factor reported in Table 7 was determined using a strict multi-modal evaluation rule: *SF*_*global*_ *=* min*(SF*_*skin*_ *, SF*_*core*_ *).* Our localized analysis revealed that the carbon composite skins maintained a high safety margin across all configurations. Consequently, the global safety factor was dictated entirely by the honeycomb core, specifically under the core shear dominant failure mode.

**FIGURE 10:**
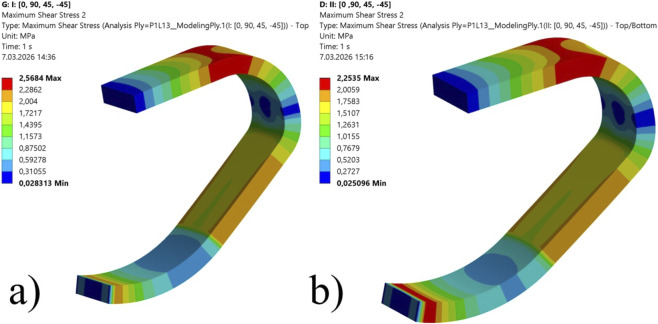
Maximum shear stress distribution within the honeycomb core dictating the dominant failure mode: Case 6, **(a)** Condition I, **(b)** Condition II.

To visualize this failure mechanism, a cloud diagram of the maximum shear stress within the core is presented in Figure 10. The contour plot reveals that the “key location” for structural failure initiation is highly concentrated at the proximal curvature.

## 4 Discussion

The present study demonstrates that a bio-inspired honeycomb sandwich architecture can substantially reduce the distal mass of a prosthetic running blade—by up to 63.9%—without compromising mechanical safety under ISO 10328 loading conditions. This mass reduction is achieved while maintaining safety factor values well above recommended thresholds, indicating that lightweighting does not inherently necessitate a loss of structural integrity. The superior stiffness-to-weight performance observed in Case 6 highlights the effectiveness of sandwich-based load redistribution mechanisms compared to conventional solid carbon laminates. These findings suggest that honeycomb core integration offers a viable structural pathway for balancing the competing demands of mass reduction, energy storage, and mechanical reliability in running prosthesis design.

The close agreement between analytical sandwich beam theory and numerical predictions indicates that the load transfer between the composite skins and the honeycomb core is accurately captured by the finite element model. This validation supports the reliability of the observed deformation and stress trends across different core configurations, suggesting that the reported performance differences arise from structural behavior rather than numerical artefacts. This level of accuracy is particularly relevant for running blade analyses, where bending response directly governs energy storage and release.

The pronounced reduction in structural mass accompanied by only a modest increase in deformation indicates that the honeycomb substitution effectively enhances the blade’s stiffness-to-weight performance (Figure 6). This trend suggests that bending rigidity is largely preserved despite substantial lightweighting, reflecting efficient load sharing between the carbon skins and the core. Similar behavior has been reported by Siddiqui et al., who showed that carbon fiber composites exhibit lower deformation sensitivity than aluminum alloys under comparable loading (Siddiqui et al., 2023a). By employing a carbon-skinned sandwich architecture, the present design avoids the excessive compliance associated with metallic blades, allowing significant mass reduction without compromising kinematic stability during stance.

The dominance of vertical flexion in the deformation response indicates that the lightweight blade preserves directional stability despite substantial mass reduction. The absence of pronounced off-axis deformation suggests that parasitic lateral or torsional motions are effectively suppressed by the sandwich architecture. Similar stabilization mechanisms have been reported by Hou et al., who emphasized the role of stiff face sheets in controlling torsional response in honeycomb panels (Georges et al., 2024). In the present design, the carbon skins appear to fulfill this role, allowing aggressive lightweighting without compromising kinematic stability during stance.

An increase in strain energy storage was also evident under Condition II loading, indicating that the sandwich architecture enhances the blade’s energy storage capacity. This behavior reflects the increased compliance introduced by the honeycomb core, allowing greater deformation under constant load and thereby promoting higher energy storage. Similar stiffness–energy trade-offs have been reported by Rigney et al., who demonstrated that reduced apparent stiffness is associated with improved energy storage and return in prosthetic components (Rigney et al., 2017). In this context, the present design leverages controlled compliance to amplify specific strain energy, enabling the blade to function as a more effective energy-storage spring despite substantial mass reduction. Such optimization aligns with performance criteria identified by Beck et al. as critical for improving functional outcomes in athletic amputees (Beck et al., 2017).

The mass reduction achieved through the sandwich architecture did not result in a critical loss of structural safety. While the solid reference blade exhibited a high safety factor of 4.11—indicating a structurally conservative but mass-inefficient configuration—the initial transition to a cellular core reduced the safety factor to 1.66, as core shear became the governing failure mechanism. Through geometric refinement of the honeycomb structure, the optimized design (Case 6) recovered this margin to a safety factor of 1.95, primarily by reducing shear stress concentrations within the core. Comparable safety factor ranges have been reported in the literature for optimized running blade designs. Ismail et al. reported a minimum acceptable safety factor of 1.29 for carbon fiber running blades under standardized loading, while Khidir et al. observed safety factors ranging between 1.72 and 2.86 across different prosthetic foot geometries (Ismail et al., 2020; Khidir et al., 2025). Within this context, the safety margin achieved in the present study reflects a deliberate balance between structural efficiency and durability, demonstrating that substantial lightweighting can be achieved without exceeding acceptable risk levels for high-impact athletic use.

## 5 Limitations

The present study focuses on the structural behavior of a prosthetic running blade within a numerical framework supported by analytical verification. Accordingly, the findings are derived from finite element simulations that assume linear elastic material behavior and idealized bonding between the composite skins and the honeycomb core. In practical manufacturing conditions, variations in skin–core adhesion quality and local imperfections may influence stress distribution and failure mechanisms. While the adopted modeling approach enables systematic evaluation of stiffness, deformation, and safety margins under standardized loading conditions, experimental validation using physical prototypes would further strengthen the assessment of the proposed design.

## 6 Conclusion

This study demonstrated that bio-inspired honeycomb sandwich architectures provide a structurally efficient alternative to conventional solid composite designs for prosthetic running blades. By redistributing load through a lightweight core–skin system, the proposed design achieved a substantial reduction in distal mass (63.9%) while preserving mechanical safety and directional stability under standardized loading conditions.

Beyond mass reduction, the optimized sandwich configuration exhibited enhanced strain energy storage, recording a 57.4% increase in energy capacity compared to the solid reference. This result indicates that controlled compliance can be effectively leveraged to improve the spring-like behavior of running prostheses without compromising structural integrity. The ability to simultaneously reduce weight, maintain a safety factor of 1.95, and increase energy storage highlights the effectiveness of honeycomb-based designs in addressing the inherently conflicting requirements of lightweight dynamics, energy return, and structural robustness.

Overall, the findings establish a clear structural rationale for integrating sandwich architectures into high-performance running prostheses and provide a practical foundation for future design strategies targeting both mechanical efficiency and user performance.

## Data Availability

The original contributions presented in the study are included in the article/supplementary material, further inquiries can be directed to the corresponding author.
